# The Revised Identification of Seniors At Risk screening tool predicts readmission in older hospitalized patients: a cohort study

**DOI:** 10.1186/s12877-022-03458-w

**Published:** 2022-11-22

**Authors:** Jane McCusker, Rebecca N. Warburton, Sylvie D. Lambert, Eric Belzile, Manon de Raad

**Affiliations:** 1 St. Mary’s Research Centre, Hayes Pavilion, 3830, suite 4720, Montreal, QC H3T 1M5 Canada; 2 Department of Epidemiology and Biostatistics, McGill University, 1020 Av. des Pins, Montréal, Montreal, QC H3A 1A2 Canada; 3 School of Public Administration, University of Victoria, 3800 Finnerty Rd (Ring Rd), Human & Social Development Building, Room A302, Victoria, BC V8P 5C2 Canada; 4 Ingram School of Nursing, McGill University, 680 Sherbrooke St W Bureau 1800, Montreal, QC H3A 2M7 Canada

**Keywords:** Older adults, Hospital, Readmission, Risk stratification, Discharge planning

## Abstract

**Background:**

The Identification of Seniors at Risk (ISAR) screening tool is a widely-used risk stratification tool for older adults in the emergency department (ED). Few studies have investigated the use of ISAR to predict outcomes of hospitalized patients. To improve usability a revised version of ISAR (ISAR-R), was developed in a quality improvement project. The ISAR-R is also widely used, although never formally validated. To address these two gaps in knowledge, we aimed to assess the ability of the ISAR-R to predict readmission in a cohort of older adults who were hospitalized (admitted from the ED) and discharged home.

**Methods:**

This was a secondary analysis of data collected in a pre-post evaluation of a patient discharge education tool. Participants were patients aged 65 and older, admitted to hospital via the ED of two general community hospitals, and discharged home from the medical and geriatric units of these hospitals. Patients (or family caregivers for patients with mental or physical impairment) were recruited during their admission. The ISAR-R was administered as part of a short in-hospital interview. Providers were blinded to ISAR-R scores. Among patients discharged home, 90-day readmissions were extracted from hospital administrative data. The primary metrics of interest were sensitivity and negative predictive value. The Area Under the Curve (AUC) was also computed as an overall measure of performance.

**Results:**

Of 711 attempted recruitments, 496 accepted, and ISAR-R was completed for 485. Of these 386 patients were discharged home with a complete ISAR-R, the 90-day readmission rate was 24.9%; the AUC was 0.63 (95% CI 0.57,0.69). Sensitivity and negative predictive value at the recommended cut-point of 2 + were 81% and 87%, respectively. Specificity was low (40%).

**Conclusions:**

The ISAR-R tool is a potentially useful risk stratification tool to predict patients at increased risk of readmission. Its high values of sensitivity and negative predictive value at a cut-point of 2 + make it suitable for rapid screening of patients to identify those suitable for assessment by a clinical geriatric team, who can identify those with geriatric problems requiring further treatment, education, and follow-up to reduce the risk of readmission.

**Supplementary Information:**

The online version contains supplementary material available at 10.1186/s12877-022-03458-w.

## Introduction

Older adults are high users of hospital services, in both emergency departments (EDs) and inpatient care. Transitional care, comprising enhanced discharge planning in coordination with community-based home-care services, can be an effective strategy to reduce rates of readmission [1, 2]. Targeting enhanced transitional care interventions by identifying patients at increased risk of readmission early during the admission may be an efficient approach to delivering these services and to optimizing patient outcomes [3].

Various tools have been developed to predict hospital readmission. In this study, we used the Identification of Seniors At Risk (ISAR), a 6-question self-report screening tool originally developed for use in the ED. ISAR is one of the most widely used risk stratification tools among seniors in the ED [4]. Test–retest reliability, content validity, concurrent validity, and predictive validity for a range of adverse outcomes (functional decline, mortality, hospitalization, community service utilization) have been established [5–9]. ISAR is used typically as step 1 of a 2-step intervention, in which it is used to target patients for a step 2 clinical assessment of geriatric problems requiring tailored intervention. Interventions targeted in this way have demonstrated positive effects on functional decline and other outcomes, including cost-effective use of resources [10–14].

Based on a meta-analysis of 32 ISAR validation studies, Galvin concluded that at the recommended cut-point of 2 +, ISAR can be a valuable clinical decision-making adjunct, essentially as a “rule-out” tool for safely discharging patients from the ED [4]. Galvin argues that the two primary metrics for a risk stratification tool that aims to predict those with adverse outcomes (the criterion for predictive validity) are the sensitivity (the proportion of those with the criterion detected by the tool) and the negative predictive value (among those with negative test results, the proportion that do not experience the criterion). Recommendations to improve the tool included exploring differential weighting of items and raising the polypharmacy threshold. There were too few studies of patients admitted to hospital for separate analysis [4]. There is a need for more research on ISAR as a risk stratification tool in the hospitalized population.

The Revised ISAR (ISAR-R) is a revision of the original ISAR made by two of the authors (RW and JM) in a Canadian ED as part of the Elder Alert geriatric assessment and management program, through a process of repeated quality improvement cycles [15]. Modifications included: setting a higher threshold for the polypharmacy question (6 + versus 4 + on original ISAR to reduce the number of patients with a positive result) and rewording two other items to improve their clarity and ease of scoring (Additional file 1). Although the ISAR-R is widely used, its psychometric properties have not been evaluated. Based on our correspondence with ISAR users, the majority are using the ISAR-R (list of users available upon request). For example, the ISAR-R has been adopted for use in the U.S. Veterans Affairs hospitals, including the GERI-VET program [14]. Note that this latter program used the ISAR-R although the original ISAR was referenced instead (personal communication, Dr. Huded).

We had the opportunity to conduct a secondary analysis of the performance of the ISAR-R in the prediction of readmission among hospitalized patients in the context of an evaluation of an enhanced discharge planning intervention. Clinical staff were blinded to the ISAR-R score. Secondary objectives were: to examine the performance of the ISAR-R in sub-groups defined by age, informant (patient or caregiver), and language; and to examine the predictive performance of individual ISAR-R items.

## Methods

### Study design

This was a secondary analysis of data collected between October 2018 and December 2019 to evaluate the implementation of an enhanced discharge education intervention (McCusker J, Beauchamp S, Lambert SD, Yaffe MJ, Meguerditchian AN, John B-T, et al: Improving transitional care for seniors: results of a patient-centered quality improvement intervention, submitted). Older adults discharged home following admission to the medical (n = 4) or geriatric (n = 2) units of two Canadian general acute-care hospitals were enrolled before (PRE) and during (POST) implementation of the intervention. Length of stay, both on the unit and overall, and 30- and 90-day readmissions were extracted from administrative data kept by the two hospitals (insufficient resources were available to use provincial administrative data). In this study we combined the PRE and POST cohorts because there was no significant change in the readmission rate (23.4% PRE to 21.9% POST, OR 0.96, 95% CI 0.81, 1.13). The study protocol and consent forms were approved by the Research Ethics Committee responsible for both hospitals. All methods were carried out in accordance with relevant guidelines and regulations.

### Recruitment

**Fig. 1:**
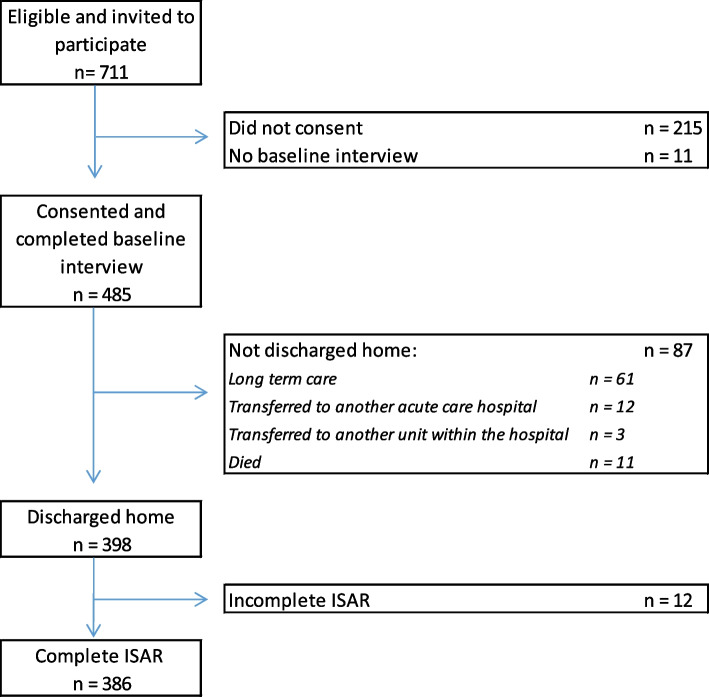
Flowchart

Patients were recruited as soon as possible after admission to the study units (including those transferred from other hospital units). Patients were enrolled at their first admission during the study period. Excluded were: patients admitted from a long-term care setting, those who were expected to be discharged to a long-term care setting, and those patients (or caregivers) who were unable to speak and read in English or French (See flowchart in Fig. 1).

Patient consent was sought for an in-hospital structured interview and a telephone follow-up after discharge to ask about discharge experiences (reported elsewhere). If clinical staff judged that the patient was not capable of informed consent (for physical or mental reasons) these patients were invited to provide assent for study staff to contact their family caregiver (unpaid family member or friend), who was invited to participate as a proxy respondent for the patient.

### Data collection

After consent, we conducted a short (5–10 min) structured interview to collect the following information on the patient: level of education; language spoken at home; country of birth (Canada vs other); receipt of local authority (CLSC) home care services; and the ISAR-R. [15] (Additional file 1). ISAR-R was used as a covariate in the main study; results were not disclosed to hospital staff. If a patient responded positively to the ISAR-R question on hospitalization during the previous 6 months, we asked whether they had been admitted to a different hospital. The answers enabled us to estimate the likely under-reporting of readmissions.

Patient data were linked to the hospital discharge databases of their admission hospital using their medical record number. The databases were used to extract hospital admissions during the 90 days after discharge, as well as discharge diagnoses during the 12 months before admission, to compute the Charlson Comorbidity Index [16], a widely-used measure of multimorbidity. (As noted above, insufficient resources were available to use provincial administrative data.)

### Sample derivation and statistical analysis

A total of 711 patients were identified by research staff as eligible and available to approach, and were invited to participate in the study. Of these, 485 (68.2%) consented and completed the in-hospital interview. Of these, 398 (82.1%) were discharged back home; 12 had one or more missing ISAR-R revised items, leaving 386 with complete ISAR-R data and formed the main analysis sample (see flowchart in Fig. 1).

To assess the representativeness of the main analysis sample (group A), we compared demographic and other characteristics with those of the following 3 groups: B—those with one or more missing ISAR-R responses (*n* = 12); C—those excluded because they had not been discharged home (*n* = 87); D—other patients aged 65 + discharged home from the same units during the study period (*n* = 2,878). The latter group was defined from hospital administrative data on all patients aged 65 + discharged home, excluding those in the structured interview sample; for those with more than one admission during the study period, we randomly selected one admission. It was not possible to identify patients in group D who would have been ineligible due to language or cognitive impairment with no proxy informant.

To compare the study sample with the excluded groups, we computed Chi-square tests for categorical variables and t-tests for continuous variables; the Kruskal–Wallis test was performed for skewed distributions [17]. The Bonferroni correction was applied at alpha 0.05 to account for multiple testing (level of significance after Bonferroni correction = 0.016) [18].

The performance of the ISAR-R on 30-day and 90-day readmission outcomes was assessed with sensitivity, specificity, positive predictive value (PPV), and negative predictive value (NPV), computed for each outcome and ISAR-R cut-point. Area Under the ROC (receiver operating characteristic) Curve (AUC) and 95% confidence intervals were computed for each outcome to estimate the overall performance of the ISAR-R score [19, 20]. For each outcome, the AUC (95% confidence interval) of the ISAR-R score was computed by age group, informant (patient vs caregiver), language and previous hospital admission at different hospital; AUC values were compared between the subgroups [20].

The associations between the 6 ISAR-R items and each outcome (30-day and 90-day readmission) were analyzed with logistic regression. For each outcome, two multivariable models were fitted; the first model included all ISAR-R items; the second model also included the covariates (hospital of index admission, service, age, previous hospitalization at different hospital, language spoken at home, informant). Odds ratios and 95% confidence intervals were computed for all ISAR-R items, the C-statistic (concordance statistic) was computed from the logistic regression model [21]. The C-statistic is equivalent to the AUC, values closer to 1 indicate better performance of the model at correctly classifying outcomes.

The performance of two shorter versions of the ISAR-R, excluding items with lower predictive value for the outcomes, was analyzed following the same approach used for the full ISAR-R. All the analyses were conducted with Stata (version 15.1, Stata Corp, College Station, TX).

## Results

In Table 1, we compare the characteristics of the study sample (group A) with those excluded or not participating in the primary study. No significant difference was observed between groups A and B (missing ISAR-R items). Those not discharged home (group C) were more often recruited at hospital 1, had longer overall hospital stays, ISAR-R was completed more often by the caregiver, and more frequently endorsed ISAR-R item 5 (memory problems). In comparison with other patients aged 65 + discharged home from the study units during the same time period (group D), the study sample was more likely to be discharged from hospital 2, to be less than 85 years old, and to have had a hospital admission in the previous 6 months.

**Table 1:** Comparison of study group with three other patient groups

Baseline	A	B	C	D			
	**Study Sample (*n* = 386)**	**Incomplete ISAR (*n* = 12)**	**Not Discharged Home (*n* = 87)**	**Not in Study (*n* = 2878)**	**Chi-square Test**		
	**%**	**%**	**%**	**%**	**A vs B**	**A vs C**	**A vs D**
Hospital					0.020	** < 0.001**	0.065
1	49.2	83.3	79.3	54.2			
2	50.8	16.7	20.7	45.8			
Unit					0.481	0.132	0.419
Geriatric	22.3	33.3	29.9	24.2			
Medical	77.7	66.7	70.1	75.8			
Age group					0.351	0.072	**0.001**
65–74	21.2	16.7	11.6	19.8			
75–84	41.5	25.0	40.7	33.5			
85 +	37.3	58.3	47.7	46.7			
Female	57.3	58.3	57.5	60.1	0.941	0.971	0.282
Previous hospital admission (6-month)**	21.2	0	26.4	15.3	0.138	0.292	**0.003**
Charlson Comorbidity Index,					0.845	0.279	0.177
0	22.8	25.0	17.2	22.5			
1	18.9	25.0	13.8	15.1			
2–3	35.2	25.0	44.8	39.6			
4 +	23.1	25.0	24.1	22.8			
Length of stay on unit, median [Q1-Q3]	6 [3–10]	5 [2–8]	6 [3–13]	5 [2–8]	0.330^a^	0.977^a^	** < 0.001**^**a**^
Length of stay overall, median [Q1-Q3]	8 [6–15]	6.5 [8.5—14.5]	21 [12—36]	7 [4–14]	0.984^a^	** < 0.001**^**a**^	** < 0.001**^**a**^
Language spoken at home				NA	0.348	0.541	NA
English	54.4	75.0	60.9				
French	25.1	8.3	21.8				
Other	20.5	16.7	17.2				
Born in Canada	59.3	66.7	64.4	NA	0.610	0.385	
Informant				NA	0.398	** < 0.001**	
Patient	84.7	75.0	67.8				
Caregiver	15.3	25.0	32.2				
Homecare services	26.5	45.5	32.9	NA	0.176	0.225	
(missing)	(5)	(1)	(2)				
Days from unit admission, mean (SD)	5.9 (5.3)	6.2 (6.0)	6.5 (11.0)	NA	0.848^b^	0.613^b^	
ISAR question*				NA			
1: Help on regular basis	39.4	50.0	39.3		0.526	0.987	
2: More help before hospitalization	45.6	30.0	52.9		0.522	0.219	
3: Previous hospitalization (6 m)	36.3	45.5	36.5		0.539	0.972	
4: Problem with vision	25.7	41.7	26.7		0.313	0.834	
5: Problem with memory	12.4	25.0	36.8		0.269	** < 0.001**	
6: Polypharmacy	61.1	44.4	52.9		0.323	0.156	
Overall score, mean (SD)	2.2 (1.4)	2.0 (1.3)	2.4 (1.5)		0.606^b^	0.202^b^	
**Outcomes:**							
30-day readmission	13.2	8.3	14.9	11.7	1.000	0.670	0.403
90-day readmission	24.9	33.3	24.1	21.0	0.506	0.886	0.081

Significant p-values are in bold font (at 0.016 after Bonferroni correction)

^a^ Kruskall Wallis Test

^b^ One way ANOVA model; NA: Not Available

^*^ ISAR question with 1 to 5 missing in Group B and C

^**^ From the administrative database

In Table 2 we present the performance characteristics of all possible cut-points of ISAR-R for the 2 outcome variables, 30- and 90-day readmission (13.2 and 24.9%, respectively). (Note than the 90-day readmissions include the 30-day readmissions.) For 90-day readmission as the outcome, at the recommended cut-point of 2 + , 65% of participants have a positive score; sensitivity is 81%, specificity is 40%, positive predictive value is 31%, and negative predictive value is 87%; AUC is 0.63 (95% CI 0.57, 0.69). Using a higher cut-point of 3 +, the number testing positive decreases markedly (65% to 39%) as does the sensitivity (81% to 51% at 90 days); specificity increases to 65%. However, the negative predictive value remains high.

**Table 2:** Performance criteria for all ISAR cut-points and readmission at 30 and 90 days after discharge (*n* = 386)

				**Predictive value**	
				** + **	**-**
**Outcomes and ISAR-R cut-points**	**Positive % (n = 386)**	**Sensitivity (%)**	**Specificity (%)**	**n (%)**	**n (%)**
**30-day readmission (51/386 = 13.2%)**		**(*n* = 51)**	**(*n* = 335)**		
1 +	91%	100%	10%	352 (14%)	34 (100%)
2 +	65%	84%	38%	252 (17%)	134 (94%)
3 +	39%	51%	63%	151 (17%)	235 (89%)
4 +	20%	31%	82%	76 (21%)	310 (89%)
5 +	4%	8%	96%	17 (24%)	369 (87%)
6	1%	2%	99%	3 (33%)	383 (87%)
AUC [95% CI]	0.63 [0.56; 0.71]				
**90-day readmission (96/386 = 24.9%)**		**(*n* = 96)**	**(*n* = 290)**		
1 +	91%	97%	11%	352 (26%)	34 (91%)
2 +	65%	81%	40%	252 (31%)	134 (87%)
3 +	39%	51%	65%	151 (32%)	235 (87%)
4 +	20%	28%	83%	76 (36%)	310 (78%)
5 +	4%	8%	97%	17 (47%)	369 (76%)
6	1%	2%	100%	3 (67%)	383 (75%)
AUC [95% CI]	0.63 [0.57; 0.69]				

*AUC* Area Under the Curve, *ISAR-R* Identification of Seniors At Risk-Revised

Table 3 shows results of analyses of AUC across sub-groups defined by age, informant, language spoken, and previous hospitalization at a different hospital. AUC varied only by age-group for 30-day readmission only, being higher in age 65–74 [0.76 (0.63, 0.89)] than in those aged 75 and over, significantly so for age 85 and over.

**Table 3:** Area under the curve (AUC) and 95% confidence interval (CI) across subgroups (*n* = 386)

**Outcomes and variables**		**n**	**AUC [95% CI]**	***p*-value**
**30-day readmission**	**Overall**	**386**	**0.63 [0.56; 0.71]**	
Age group:				
	65–74	82	0.76 [0.63; 0.89]	(reference)
	75–84	160	0.59 [0.45; 0.73]	0.069
	85 +	144	0.58 [0.46; 0.70]	**0.031**
Informant:				
	Patient	327	0.64 [0.56; 0.72]	(reference)
	Caregiver	59	0.54 [0.37; 0.71]	0.279
Language spoken at home				
	English	210	0.66 [0.56; 0.76]	(reference)
	French	97	0.64 [0.49; 0.79]	0.826
	Other	79	0.53 [0.38; 0.69]	0.178
Previous admission to different hospital				
	No admission	246	0.63 [0.54; 0.72]	(reference)
	Same hospital	121	0.56 [0.42; 0.70]	0.387
	Different hospital	19	0.69 [0.39; 0.99]	0.710
**90-day readmission**	**Overall**	**386**	**0.63 [0.57; 0.69]**	
Age group:				
	65–74	82	0.67 [0.54; 0.81]	(reference)
	75–84	160	0.65 [0.55; 0.75]	0.795
	85 +	144	0.58 [0.48; 0.68]	0.272
Informant:				
	Patient	327	0.62 [0.56; 0.69]	(reference)
	Caregiver	59	0.63 [0.48; 0.78]	0.919
Language spoken at home				
	English	210	0.61 [0.53; 0.69]	(reference)
	French	97	0.69 [0.57; 0.81]	0.286
	Other	79	0.63 [0.47; 0.78]	0.884
Previous admission to different hospital				
	No admission	246	0.62 [0.54; 0.70]	(reference)
	Same hospital	121	0.51 [0.40; 0.61]	0.098
	Different hospital	19	0.69 [0.39; 0.99]	0.653

Significant p-values are in bold font

Table 4 shows the logistic regression models for each ISAR-R item and the two outcomes. Multivariable model 1 includes only the 6 ISAR-R items; model 2 also includes the covariates, hospital, unit, age, previous hospitalization at a different hospital, language, and informant. Previous hospitalization is the only significant predictor of 30-day readmission in the multivariable models 1 and 2. In model 1 for 90-day readmission, item 2 (more help needed than before admission) is also significant; in model 2, only item 4 (problem with vision) is significant. Notably, items 1 (need for help on a regular basis) and 6 (polypharmacy) show little association (OR less than 1.5) with either outcome, and item 5 (problem with memory) has an OR greater than 1.5 only in the univariate model for 90-day readmission.

**Table 4:** Logistic regression models for individual ISAR-R items and readmission outcomes (*n* = 386)

Outcomes and ISAR items	Logistic regression for each outcome					
	**Univariate models**		**Multivariable model 1**		**Multivariable model 2**	
	**OR**	**[95% CI]**	**OR**	**[95% CI]**	**OR**	**[95% CI]**
**30-day readmission:**						
1- Help on regular basis	1.11	[0.62; 2.00]	0.89	[0.48; 1.67]	0.78	[0.40; 1.51]
2- More help before hospitalization	**1.83**	**[1.02; 3.29]**	1.68	[0.92; 3.09]	1.65	[0.89; 3.04]
3- Previous hospitalization (6 m)	**1.85**	**[1.03; 3.32]**	1.69	[0.92; 3.12]	1.52	[0.73; 3.19]
4-Problem with vision	**2.85**	**[1.56; 5.18]**	**2.84**	**[1.53; 5.25]**	**2.85**	**[1.53; 5.32]**
5-Problem with memory	0.89	[0.36; 2.20]	0.70	[0.27; 1.79]	0.61	[0.23; 1.64]
6-Polypharmacy	1.28	[0.69; 2.35]	1.09	[0.58; 2.08]	1.17	[0.61; 2.24]
C-statistic [95% CI]			0.67 [0.58; 0.76]		0.68 [0.59; 0.77]	
**90-day readmission:**						
1- Help on regular basis	1.16	[0.73; 1.84]	0.94	[0.57; 1.54]	0.87	[0.52; 1.47]
2- More help before hospitalization	**1.86**	**[1.17; 2.95]**	**1.66**	**[1.03; 2.66]**	1.62	[0.99; 2.62]
3- Previous hospitalization (6 m)	**2.18**	**[1.37; 3.47]**	**1.97**	**[1.22; 3.18]**	1.55	[0.87; 2.89]
4-Problem with vision	**1.75**	**[1.07; 2.88]**	1.64	[0.98; 2.75]	**1.71**	**[1.01; 2.89]**
5-Problem with memory	1.69	[0.89; 3.20]	1.44	[0.74; 2.81]	1.39	[0.68; 2.82]
6-Polypharmacy	1.25	[0.78; 2.01]	1.08	[0.66; 1.78]	1.12	[0.67; 1.86]
C-statistic [95% CI]			0.65 [0.58; 0.72]		0.68 [0.61; 0.75]	

For each outcome: model 1 includes all the ISAR-R items; model 2 include all the ISAR-R items plus covariates (hospital of index admission, service, age, previous admission at different hospital, language, informant)

*OR* Odds Ratio, *CI* Confidence Interval, Significant ORs (*p*-value < 0.05) are in bold font

Additional file 2 shows the performance characteristics of two shorter versions of ISAR-R comprising items 2-4 and 2-5, respectively. Values for the AUC are somewhat higher than for the 6-item ISAR-R (0.64-0.66).

## Discussion

The revised ISAR-R evaluated in this study is widely used, although only previously studied in a quality improvement context [15]. Thus, our results provide preliminary evidence of its predictive validity, as well as adding to the small number of studies of ISAR when used in hospitalized patients. Overall, the results are similar to those of previous studies of ISAR. The main advantages of the ISAR-R are the higher threshold for polypharmacy, reducing the number of patients who will screen positive, and the more intuitive phrasing and scoring of questions, facilitating administration and scoring [15].

The main changes made in the ISAR-R were in two items: problems with vision and polypharmacy (Additional file 1). The vision question was rephrased to avoid reverse scoring: the original question was “In general, do you see well?” with a “no” response scored as 1. In the revised ISAR-R, the question became “In general, do you have serious problems with your vision that cannot be corrected with glasses?”, with a “yes” response scored as 1. This question was one of the most important predictors of outcome in the current study, particularly for 30-day readmissions, with an unadjusted OR of 2.85 and OR adjusted for other ISAR-R items of 2.84. For 90-day readmission, the unadjusted and adjusted ORs were 1.75 and 1.64, respectively. For comparison, in a previous study the unadjusted and adjusted ORs for 6 month top decile of hospital days were 1.08 and 1.22 [7]. It appears that the revised vision question may improve performance as well as facilitating ISAR-R administration.

As regards polypharmacy (ISAR-R item 6), the threshold was increased from 4 + medications in the original ISAR to 6 + because the increase over time in the number of medications used by older people increased the number of patients with positive screens [15, 22, 23]. In our previous research, the polypharmacy question (4 + medications) had unadjusted and adjusted ORs of 1.43 and 1.57, respectively [7], compared to 1.25 and 1.08 in the current study using the 6 + threshold. It appears that the revised question may be less predictive of readmission than the original one. Furthermore, a high proportion of patients (61%) answered yes to this question. Possibly, use of a higher threshold may be more discriminating. Another item that performed poorly in our study is item 1 on needing help on a regular basis. In our original research on ISAR and prediction of 6 months hospitalization, unadjusted and adjusted ORs were 1.36 and 1.78 [7], respectively, versus 1.16 and 0.94 in current study. Interestingly, although items 1 and 6 did not contribute meaningfully to the prediction of readmission, the elimination of these items does not appreciably improve the performance of the tool in predicting readmission. Further research on prediction of functional decline and other outcomes by these items is warranted.

ISAR-R performed better in those aged 65–74 than in older age groups in predicting 30-day readmission, perhaps due to better cognitive functioning and more accurate reporting. The original ISAR performed similarly in different age groups in predicting various outcomes [6, 7]. As in previous studies of ISAR, ISAR-R performed similarly when using patient or proxy informants and in different language groups. These properties enhance the feasibility of using ISAR-R in diverse patient populations.

Only three previous studies, to our knowledge, have investigated the performance of the original ISAR in predicting readmission among hospitalized patients [24–26]. Our study found that ISAR-R had sensitivity, NPV, and specificity values within the ranges previously reported: high sensitivity (76–86%) and NPV (79–90%) but modest specificity (33–44%) [24–26]. The AUC was reported in only one of these studies as 0.60 (95% CI 0.55, 0.65) [26]; our estimate was somewhat higher (0.63, 95% CI 0.57, 0.69). These results indicate modest predictive performance of the tool, consistent with meta-analyses [4, 27].

While these results do not justify the use of either ISAR or ISAR-R as a stand-alone clinical prediction tool, these tools can be used as adjuncts in clinical decision-making. The high values of sensitivity indicate that the great majority of those at high risk of readmission will be detected; the high negative predictive value make the tool useful to rule out patients at risk of readmission, useful properties in a short, easily-administered tool. Nevertheless, the low specificity implies that there are substantial false-positives that can be identified at a second step clinical assessment of geriatric problems. For example, in a previous trial of a two-step ED intervention for older adults in the ED, using a standardized nursing assessment, 61.2% of those scoring ISAR 2 + were found to have one or more new or uncompensated geriatric problems [10]. Centers wishing to reduce the number of patients testing positive (and the false-positives) can increase the cut-point to 3 + . This higher cut-point reduces the sensitivity but maintains a high negative predictive value.

The search for better screening tools has been pursued, with results varying by patient population and tools compared. A meta-analysis of screening tools to predict functional decline in hospitalized older adults found 3 tools, including ISAR, worthy of further investigation [28]. In a subsequent head-to-head comparison, ISAR was found to perform similarly to or better than the other two tools and was judged to be the easiest to use in clinical practice [29]. Three other studies to our knowledge have compared ISAR directly to other screening tools in the same patient population when administered after admission to hospital [25, 30, 31]. Different outcomes were examined (functional decline, readmission). In general, all tools including ISAR had modest predictive performance. [Note that the 4-item so-called ISAR-HP was not derived from the ISAR and is based on different items [32]].

## Limitations

Several study limitations should be highlighted. First, the study was a secondary analysis, limited by the data collected in the main study. Thus, we were not able to compare the performance of the ISAR-R to that of the original ISAR. Also, we were not able to follow patients discharged to LTC or alternative institutions (*n* = 87). Table 1 show that this group differs from the main sample in several respects (longer hospital stay, informant was more often the caregiver, more self-reported memory problems). A second limitation is that the study sample is not representative of all patients discharged home from the same study units during the same period: patients aged 85 + , those with a previous hospital admission, and those with longer hospital stays are over-represented (Table 1). Third, readmissions were defined from administrative data at the same hospital as the index admission. However, only a small minority of patients reported admissions to different hospitals; stratification for this variable did not significantly affect the AUC (Table 3).

## Conclusions

This study provides preliminary evidence that the ISAR-R performs at least as well as the original ISAR in prediction the prediction of readmission among older patients following admission to hospital. This will be reassuring for the many institutions which have adopted the ISAR-R as a step 1 of a 2-step screening program, although further research is needed. It may be a valuable clinical tool to help stratify patients into lower and higher-risk groups to guide interventions that aim to reduce readmission. The advantages of the ISAR-R over the original ISAR are: 1) the more intuitive phrasing and scoring of the questions and 2) reduction of the number of patients that screen positive through revision of the polypharmacy threshold (as in the original QI study) [15]. Further research is recommended in three areas: first, the tool’s performance in predicting different outcomes (e.g., functional decline, mortality, and nursing home admission); second, optimal wording of ISAR questions for different populations; and third, how best to implement screening in different clinical contexts.

## Supplementary Information

**Additional file 1. **ISAR tool: Original and revised versions 

**Additional file 2. **Performance criteria for two different versions of ISAR-R tool;30 and 90 days readmission outcomes (*n*=386) 

## Data Availability

The datasets used and analysed during the current study are available from the corresponding author on reasonable request. Data are not available publicly because of privacy.
