# Supplementary material for: The Revised Identification of Seniors At Risk screening tool predicts readmission in older hospitalized patients: a cohort study
Source: BMC Geriatr. 2022 Nov 22;22:888. doi: 10.1186/s12877-022-03458-w (PMC9682664; doi:10.1186/s12877-022-03458-w)
Supplement: Supplementary file 1 — Additional file 1. ISAR tool: Original and revised versions [file 12877_2022_3458_MOESM1_ESM.docx]

Appendix A: ISAR tool: Original and revised versions
